# Laboratory Diagnosis of Antiphospholipid Syndrome: Insights and Hindrances

**DOI:** 10.3390/jcm11082164

**Published:** 2022-04-13

**Authors:** Arne Vandevelde, Katrien M. J. Devreese

**Affiliations:** 1 Department of Diagnostic Sciences, Ghent University, 9000 Gent, Belgium; arne.vandevelde@ugent.be; 2 Coagulation Laboratory, Ghent University Hospital, 9000 Gent, Belgium

**Keywords:** anti-β2 glycoprotein I antibodies, anticardiolipin antibodies, antiphospholipid antibodies, antiphospholipid syndrome, interference, lupus anticoagulant, non-criteria antiphospholipid antibodies

## Abstract

Diagnosis of antiphospholipid syndrome (APS) requires the presence of a clinical criterion (thrombosis and/or pregnancy morbidity), combined with persistently circulating antiphospholipid antibodies (aPL). Currently, laboratory criteria aPL consist of lupus anticoagulant (LAC), anticardiolipin antibodies (aCL) IgG/IgM, and anti-β2 glycoprotein I antibodies (aβ2GPI) IgG/IgM. Diagnosis and risk stratification of APS are complex and efforts to standardize and optimize laboratory tests have been ongoing since the initial description of the syndrome. LAC detection is based on functional coagulation assays, while aCL and aβ2GPI are measured with immunological solid-phase assays. LAC assays are especially prone to interference by anticoagulation therapy, but strategies to circumvent this interference are promising. Alternative techniques such as thrombin generation for LAC detection and to estimate LAC pathogenicity have been suggested, but are not applicable yet in routine setting. For aCL and aβ2GPI, a lot of different assays and detection techniques such as enzyme-linked immunosorbent and chemiluminescent assays are available. Furthermore, a lack of universal calibrators or standards results in high variability between the different solid-phase assays. Other non-criteria aPL such as anti-domain I β2 glycoprotein I and antiphosphatidylserine/prothrombin antibodies have been suggested for risk stratification purposes in APS, while their added value to diagnostic criteria seems limited. In this review, we will describe laboratory assays for diagnostic and risk evaluation in APS, integrating applicable guidelines and classification criteria. Current insights and hindrances are addressed with respect to both laboratory and clinical implications.

## 1. Historical Perspective into Antiphospholipid Antibodies

In the 1950s, multiple cases were reported about the presence of a circulating anticoagulant that inhibited the conversion of prothrombin to thrombin in patients with systemic lupus erythematosus (SLE), resulting in prolonged clotting times [1]. These prolongations could not be corrected by mixing patient blood or plasma with normal blood or plasma [1,2,3,4,5]. Feinstein and Rappaport introduced the term ‘lupus anticoagulant’ (LAC) in 1972 for a circulating anticoagulant that blocked the prothrombin activator complex (factor Xa (FXa), factor V, calcium, and phospholipids), resulting in prolonged activated partial thromboplastin time (aPTT) and prothrombin time (PT) [6]. It was called ‘anticoagulant’ because of the in vitro effect on coagulation tests and the initial cases described with hemorrhagic diathesis [6,7]. In the same period, it was already suspected that the presence of LAC was not often associated with bleeding [1,7,8]. It was named after ‘lupus’ as this was the most observed coagulation abnormality in SLE, although also present in non-SLE disorders [1,7]. In the 1980s, it became more and more clear that ‘lupus anticoagulant’ was a misnomer as it appeared to be associated in vivo with thrombosis rather than bleeding, paradoxically to its in vitro effect on clotting times [9,10].

Along with the LAC phenomenon finding, the association of false-positive syphilis testing in patients with other infectious and autoimmune diseases drew attention [11,12,13]. It was estimated that up to 20% of patients with SLE had positive serological syphilis testing without history or clinical signs of syphilis [13]. The antigen responsible for the serologic reaction appeared to be a phospholipid named cardiolipin [14,15,16]. In 1957, Laurell and Nilsson described two patients with presence of a circulating anticoagulant combined with a false-positive syphilis test. Both antibodies causing the in vitro anticoagulant effect and the positive syphilis test were located at the same region on the electrophoretic separation pattern, suggesting the relationship between the two phenomena [17]. In the 1970s, more studies confirmed the association of false-positive syphilis tests and LAC presence [8,18]. It was hypothesized that antibodies against cardiolipin reacting in the syphilis tests were identical to those causing LAC effect by interfering with phospholipids in the prothrombin activator complex [19].

In 1983, Harris et al. reported the development of a solid-phase radioimmunoassay for detecting antibodies against cardiolipin. They observed a strong correlation between anticardiolipin antibodies (aCL) and LAC, and a significant correlation between high aCL IgG/IgM levels and thrombosis [20]. In the same journal issue, Graham Hughes described the association between presence of LAC, aCL and a clinical picture of abortions, thrombosis, and neurologic manifestations [19]. The clinical description was initially called anticardiolipin syndrome, but nomenclature changed to antiphospholipid syndrome (APS) later in the 1980s, when it became clear that antibodies did not only react with cardiolipin, but also with other phospholipids [21]. To date, the detection of LAC relies on its ability to prolong phospholipid-dependent coagulation assays, as already described in the first guidelines on LAC detection by the International Society on Thrombosis and Haemostasis (ISTH) [22], and followed in the consecutive updates [23,24,25], as well as in guidelines of the British Society for Haematology (BSH) [26,27,28], and Clinical and Laboratory Standards Institute (CLSI) [29]. By the beginning of the 1990s, it became clear that aCL require the presence of a cofactor, β2-glycoprotein I, for interaction with cardiolipin [30,31,32]. Development of enzyme-linked immunosorbent assays (ELISAs) detecting anti-β2-glycoprotein I antibodies (aβ2GPI) soon followed, showing a good correlation with aCL and association with thrombosis [33,34].

## 2. Classification and Diagnostic Criteria

Since it became clear that antiphospholipid antibodies (aPL) were significantly associated with vascular thrombosis and pregnancy morbidity, the need for consensus criteria for APS resulted in 1999 in preliminary classification criteria for definite APS, named the Sapporo criteria [35]. As research data and clinical experience grew in the following years, updated classification criteria were published in 2006, named the Sydney criteria (Table 1). Patients were classified as having APS when a clinical event occurs (vascular thrombosis and/or pregnancy morbidity) along with at least one positive laboratory criterion. Laboratory criteria were defined as the presence of LAC, aCL IgG/IgM in medium to high titer, or aβ2GPI IgG/IgM higher than the 99th percentile, persistently present for at least 12 weeks [36]. The persistent presence of antibodies increases specificity for APS as non-pathogenic, reactive antibodies through infection or drugs are usually transient [37,38]. While these criteria were not developed for diagnosing APS, but for facilitating and standardizing clinical research, they are often used as diagnostic criteria in clinical practice [39]. Besides the criteria currently regarded as classification criteria for APS, other manifestations such as thrombocytopenia, autoimmune hemolytic anemia, livedo reticularis, neurologic manifestations, nephropathy, and valvular heart disease are associated with presence of aPL [40,41,42,43]. According to the current guidelines, LAC, aCL IgG/IgM, and aβ2GPI IgG/IgM are considered as laboratory criteria for APS [44]. Patients with high clinical suspicion of having APS, but without presence of criteria aPL, are suggested to have “seronegative APS” (SNAPS). These patients may be positive for so-called non-criteria aPL or be negative for the criteria aPL through insufficient sensitivity of the assays [40,45]. Currently, a new international, multidisciplinary initiative is undertaken for development of new, comprehensive APS classification criteria. The proposed candidate laboratory criteria include current criteria antibodies (LAC, aCL IgG/IgM, and aβ2GPI IgG/IgM) only. Definition of laboratory persistence, antibody titer cut-off, and use of age-related cut-offs will be described in further publications of this collaborative initiative [46].

## 3. Lupus Anticoagulant

### 3.1. Test Procedure

#### 3.1.1. Choice of Assay

**Figure 1:**
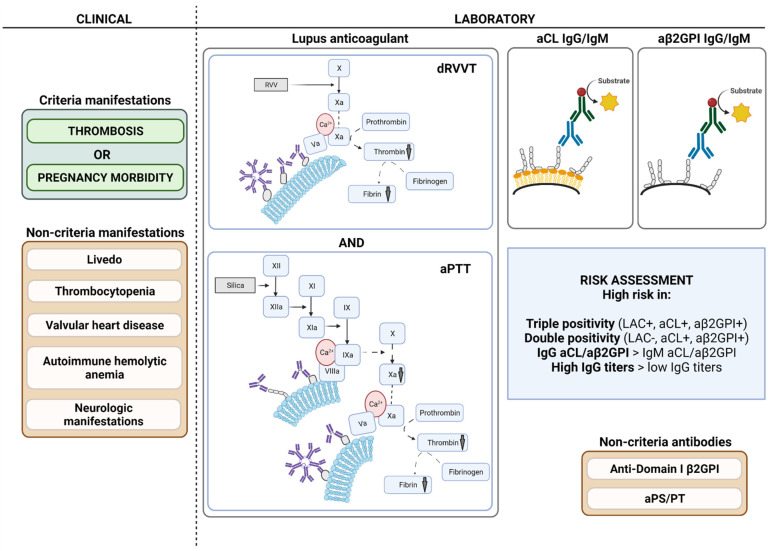
Diagnosis of APS is based on presence of at least one clinical criterion, whether or not accompanied by other non-criteria manifestations, and presence of at least one laboratory criterion over at least 12 weeks. Laboratory criteria consist of lupus anticoagulant (LAC), anticardiolipin (aCL) IgG/IgM antibodies, and anti-β2 glycoprotein I (aβ2GPI) IgG/IgM antibodies. LAC is measured by applying two phospholipid-dependent coagulation tests, the diluted Russell’s viper venom time (dRVVT) and LAC-sensitive activated partial thromboplastin time (aPTT). The effect of LAC is schematically demonstrated. In dRVVT, factor X is directly activated by Russell’s viper venom (RVV), consequently activating prothrombin to thrombin in a phospholipid-, calcium-, and activated factor V-dependent way. Thrombin will further convert fibrinogen into fibrin, which can be detected mechanically or optically. In aPTT, factor XII is activated by a contact activator (silica) which initiates a sequence of activation of factor XI, IX, X, prothrombin, and conversion of fibrinogen to fibrin. Both activation of factors IX and X depends on phospholipids. Antiphospholipid antibodies present in the patient plasma sample will compete with coagulation factors for phospholipids through binding with phospholipid-bound cofactors such as β2-glycoprotein I, resulting in a prolonged clotting time. Positive screening assays are followed by mixing and confirmation tests. In aCL assays, a solid phase is coated with cardiolipin which forms a complex with β2GPI and aβ2GPI assays are coated with β2GPI. Patient aCL or aβ2GPI can bind to the antigen on the solid phase. Subsequent binding of reagent anti-human IgG/IgM antibody labeled with conjugate leads to conversion of a substrate into a measurable product. Risk for clinical events is highest for patients with triple- and double-positive profiles. Higher risk is also observed for IgG aCL/aβ2GPI compared to IgM, and higher IgG titers are more associated with clinical events compared to lower positive titers. Non-criteria antiphospholipid antibodies such as anti-domain I β2GPI IgG and antiphosphatidylserine/prothrombin antibodies are currently not part of the laboratory criteria, and are currently not advised to be measured in routine practice. Created with BioRender.com.

The term LAC comprises a heterogeneous group of autoantibodies, responsible for prolongation of phospholipid-dependent coagulation tests. LAC can be detected by different phospholipid-dependent coagulation tests. The most recent update of the ISTH guidelines on LAC detection recommends parallel testing of the dilute Russell’s viper venom time (dRVVT) and aPTT [25]. dRVVT is more specific for LAC, while aPTT is more sensitive, although strongly dependent on the reagent used. Both assays are complementary as aPL do not always react in both test systems [47]. The dRVVT assay is based on direct activation of factor X by an enzyme present in the venom of Russell’s vipers. aPL in patient plasma will react with phospholipid components of the reagent through cofactors and prolong the dRVVT by decreased activity of the prothrombin activator complex (Figure 1) [48]. The aPTT assay is based on activation of the contact (intrinsic) pathway. Analogous to the dRVVT assay, aPL inhibit phospholipid-dependent steps in the aPTT coagulation pathway (Figure 1). Selection of adequate reagents for LAC testing purposes is of great importance as numerous different reagents are available, especially for aPTT, with different sensitivity for LAC detection [49]. Two key points in aPTT reagent selection are choice of activating agent, and phospholipid composition and concentration [25]. It is recommended to select an aPTT assay with silica as activator, while ellagic acid could be considered as an alternative in aPTT reagents with comparable sensitivity for LAC [25,50]. As an alternative for aPTT, the silica clotting time (SCT), a phospholipid-dependent coagulation test using silica as activator can be used for LAC testing [25,51,52,53,54]. dRVVT assays are specifically commercialized for LAC testing and show less variability compared to aPTT assays. However, it was demonstrated that dRVVT reagents can differ in LAC sensitivity and susceptibility to vitamin K antagonist (VKA) anticoagulation treatment [55,56,57,58].

Besides dRVVT and aPTT, other phospholipid-dependent coagulation assays are not recommended due to variability in reagent composition, poor reproducibility or limited commercial availability [25]. The dilute prothrombin time (dPT) is included in the BSH guidelines as an alternative for aPTT [27] and CLSI guidelines for second-line testing [29]. The dPT shows good sensitivity in detecting LAC, but its use is hampered by considerable variability in reagents [25,59,60]. The kaolin clotting time (KCT) is based on activation of the intrinsic pathway by kaolin as contact activator and mainly differs from aPTT by absence of exogenous phospholipid source [61]. While KCT was used frequently in the past, it has been largely abandoned because of standardization issues, a lack of confirmatory tests, and incompatibility with certain analyzers using optical clot detection [24,25,52,62,63]. The Taipan snake venom time (TSVT) combined with ecarin time (ET) is a promising assay for LAC assessment [64,65]. It was recently evaluated in a multicenter setting showing good sensitivity for LAC detection compared to aPTT and dRVVT with less interference of oral anticoagulation treatment. In the TSVT assay, oscutarin C from venom of the Coastal Taipan viper activates prothrombin into thrombin in a phospholipid- and calcium-dependent, but Factor V-independent way [65,66]. ET contains venom from the Indian Saw-Scaled viper in which ecarin activates prothrombin independently from any cofactor such as phospholipids [67]. TSVT/ET combination can be used as screening/confirmation assay as ET is phospholipid independent [65]. TSVT/ET tests are less affected by VKA and anti- FXa direct oral anticoagulants (DOACs), and may be a solution for LAC testing in anticoagulated patients. More evidence from collaborative studies with standardized assays is needed before their general use can be advised [68].

Performance of LAC assays has to be validated or verified before implementation in routine practice. Part of the verification process includes testing of samples with known LAC potency. Ideally, properly stored and well-characterized LAC samples are used in the evaluation process [25,29,69]. This is especially important for evaluating aPTT-based assays as large differences in sensitivity between reagents are observed [49]. 

#### 3.1.2. Analytical Procedure

LAC measurement traditionally consists of a three-step procedure performed on platelet-poor citrated plasma: screen, mix, and confirm [25,27]. Platelet-poor plasma (<10,000 thrombocytes/µL) is required to avoid false-negative results due to interaction of platelet-derived phospholipids and aPL. Expressing results of the patient plasma as a normalized ratio through dividing the patient clotting time result by the pooled normal plasma (PNP) result reduces inter-laboratory and between-run variation [25]. To also compensate for the day-to-day variation, analyzing a PNP in each run is preferred over the use of the mean of a reference interval determined per lot of reagent as advised in the CLSI guidelines [29].

The screening step comprises testing with both dRVVT and aPTT reagents at low phospholipid concentration. As factor deficiency or inhibitors other than LAC can cause a positive screening assay, it has to be followed by a mixing and confirmation step for the assays positive in the screening procedure. This step-wise procedure can reduce costs, as this avoids unnecessary performance of the mixing and confirmation step if the screening step is not prolonged, although in daily practice, paired assays screening and confirmation assays are often performed simultaneously, followed by the mixing test in a next step. A positive screening test is followed by a mixing test and confirmatory test, irrespective the result of the mixing test [25].

In the confirmatory step, an excess of anionic phospholipid is added to the test reagent, which reduces or neutralizes the inhibitory effect of antibodies causing the prolonged clotting time. In paired tests, usually dRVVT, where screen and confirm assays are performed in parallel, the result of the confirmation step is expressed as normalized LAC ratio [(screen patient)/(screen PNP)]/[(confirm patient)/(confirm PNP)], or as normalized percentage correction [(screen patient)/(screen PNP) − (confirm patient)/(confirm PNP)]/[(screen patient)/(screen PNP)] ∗ 100 [25,29]. 

In the mixing step, the screening assay is performed on a mixture of 1:1 patient plasma and PNP (screen mix). The mixing test is expressed as the normalized ratio [(screen mix)/(screen PNP)]. When the clotting time in the confirmatory assay is prolonged, meaning that no full correction is observed by adding excess phospholipids, an additional mixing step with the confirmatory reagents (confirm mix) should be performed [25,29]. The ratio of this mixing test is calculated as [(screen mix)/(screen PNP)]/[(confirm mix)/(confirm PNP)]. This ratio is more robust and less affected by interference of congenital or acquired factor deficiencies, or by very strong LAC activity that is not corrected by the excess phospholipid in the confirmatory analysis [25].

Integrated assays exist that perform all three steps in one procedure. In these assays, both screening and confirmation tests are performed concomitantly on patient plasma mixed with PNP and results are mostly expressed as the difference (delta value) between both tests. Paired tests perform both screening and confirmation in one procedure, meaning that the mixing step still needs to be performed when the screening step is positive [25].

#### 3.1.3. Cut-Off Values

LAC interpretation requires determination of adequate cut-off values to determine positivity in all three steps. Preferably, laboratories determine in-house cut-off values using a sufficiently large population of healthy reference individuals (at least 120), determining the cut-off as 99th percentile after outlier rejection [25]. Using a parametric approach, as is described in the CLSI guidelines, assumes a Gaussian distribution of the results which should be checked by inspection of the histogram [29]. However, multiple reports have shown that the distribution of LAC results does not adhere to the Gaussian probability distribution [70,71]. Cut-off values based on a 99th percentile increase specificity, but reduce sensitivity compared to the parametric approach, which applies the 97.5th percentile [25]. Increased specificity is warranted to decrease false positives, considering that decreased sensitivity is partially intercepted by use of two screening assays in LAC assessment.

The large number of 120 normal individuals to calculate adequate cut-off values hampers many laboratories, as illustrated in a survey from the ISTH showing that only 12% of participants who consider the 99th percentile use at least 120 healthy volunteers [72]. An approach requiring fewer volunteers, is the transference of cut-off values suggested by the manufacturer. This assumes that suggested cut-offs are based on a large healthy reference population with adequate demographics, a correct statistical method, and a correct reagent–instrument combination. When these assumptions are met, manufacturer cut-off values have to be verified before transference by testing 20 healthy volunteers representing the local population demographics. After outlier rejection and replacement with new healthy volunteer results, the outlier-free population results are compared with the suggested cut-off value. CLSI guidelines recommend that no more than two samples should fall outside the limits [73]. When more than two results exceed the cut-off value, a new set of healthy volunteer samples has to be analyzed. After outlier rejection, suggested cut-off values can be transferred when a maximum of two results in the new set of samples are above the cut-off value. CLSI guidelines also provide the option for transference of cut-off values established in other laboratories; however, this is not recommended as significant inter-laboratory variation is observed, even when using the same reagent or analyzers [25,70,74].

### 3.2. Interferences and Limitations

Inherent to the test principle of phospholipid-based coagulation assays, LAC testing is prone to interferences.

C-reactive protein interferes in vitro with aPTT testing through its affinity for phospholipids, leading to false-positive results of the LAC test. While this effect was not observed for the dRVVT assay based on in vitro experiments, this could differ between reagents and effect of CRP cannot be excluded for reagents using a different phospholipid composition [75,76]. Increased factor VIII (FVIII) coagulant activity is associated with shorter aPTT clotting times, and could lead to false-negative LAC aPTT screening assays [77]. dRVVT screening is not influenced by FVIII levels as factor X is directly activated by Russell’s viper venom. Increased levels of FVIII can be observed during pregnancy, surgery, inflammation, malignancy, and other conditions [78]. LAC is often found to be positive during inflammatory conditions, without clear association with a clinical APS phenotype, recently highlighted in patients with coronavirus disease 2019 [79,80,81]. Indeed, it is known from viral and bacterial infections that post-infectious presence of LAC usually is transient and not accompanied by the clinical APS phenotype [38]. Certain drugs (e.g., antibiotics, antiarrhythmics, and chlorpromazine) and to a lesser extent vaccines (e.g., against hepatitis B virus) are also found to be associated with LAC activity [38]. Furthermore, in the acute setting of thrombosis, increased FVIII levels can lead to false-negative LAC assessment, while increased CRP can lead to false-positive LAC testing. Therefore, it is not recommended to assess LAC status during the thrombotic event or in patients with acute inflammation. Retesting of patients with LAC positivity, at least 12 weeks after the initial finding, is an important strategy in avoiding misclassification of patients with transient LAC [25].

Anticoagulation treatment complicates LAC testing and interpretation by prolonging aPTT and dRVVT. LAC testing during anticoagulation treatment is discouraged [25,27,29,68], although it is not always desirable to postpone LAC analysis until treatment cessation [68]. Testing for LAC during anticoagulant treatment can be useful in view of the duration of therapy and choice of anticoagulant based on the aPL profile [82]. However, interruption of anticoagulation treatment for LAC testing could potentially increase the thrombotic risk for the patient, especially shortly after a thrombo-embolic event.

VKAs cause prolongation of aPTT and dRVVT through production of incomplete coagulation factors by inhibition of vitamin K-dependent gamma carboxylation of factors II (prothrombin), VII, IX, and X [83]. This acquired factor deficiency can lead to false-positive interpretation of LAC testing, especially in the screening step, and false negative in the mixing step. However, certain dRVVT reagents appear to be less prone to VKA treatment, or false positivity can be overcome by applying a higher screen/confirmation ratio cut-off [55,57,84], making the interpretation of LAC even more complicating. CLSI, BSH, and the earlier 2009 ISTH guidelines include the possibility of 1:1 dilution of patient plasma into PNP to correct for the acquired factor deficiency [24,27,29]. This is not advised since LAC activity is hereby also diluted, leading to potential false-negative results; interpretation remains complicated, and the degree of correction depends on the reagents used [68,85,86]. Because of these issues, the most recent ISTH guidelines do not advise on predilution of samples for LAC testing in presence of VKAs [25,68]. In 2021, a large multicenter study demonstrated good sensitivity and specificity of the TSVT/ET test for detecting LAC in VKA anticoagulated patients [65]. However, because of limited commercial availability, it might not be feasible to include TSVT/ET testing in routine practice yet [25,65,68]. To overcome the interference with VKA, treatment could be temporarily interrupted, switching patients to low-molecular-weight heparin (LMWH) which shows no LAC interference in the dRVVT and aPTT test system at therapeutic levels [87]. This procedure does require a long bridging period to allow wash-out of the VKA, frequent monitoring of international normalized ratio (INR) to assess recuperation from VKA effect, and carries along a potentially higher bleeding risk [68].

Unfractionated heparin (UFH), LMWH and heparinoids mainly interfere by indirectly inhibiting thrombin and FXa action [88]. Most dRVVT and some LAC-specific aPTT reagents contain heparin-neutralizing agents, heparinase or hexadimethrine bromide (Polybrene), quenching the effect of heparin in vitro. Manufacturers should specify until which anti-FXa activity level heparin is quenched, mostly being approximately 0.8–1.0 IU/mL. Moreover, when applying the three-step algorithm of screening, mixing, and confirming, no false-positive LAC tests are observed [87]. Supratherapeutic levels should be ruled out by anti-FXa activity testing along with LAC testing [25,68].

DOACs directly inhibit thrombin (e.g., dabigatran) or FXa (e.g., apixaban, betrixaban, edoxaban, and rivaroxaban) [89], with various effects on coagulation tests, even at trough levels, leading to both false-negative and false-positive LAC interpretation [90,91,92,93,94,95]. Parenteral direct thrombin inhibitors are associated with false LAC positivity (e.g., argatroban) [96]. Adsorption techniques have been described to overcome DOAC interference in vitro. It was demonstrated that adding activated carbon/charcoal to citrated plasma samples removes DOAC from the sample and avoids interference for PT, aPTT, dRVVT, and SCT assays without significant interference on the coagulation assay itself [97]. Current hypotheses assume that activated carbon products adsorb small, neutral or positively charged anticoagulants such as DOACs in their pores [98,99]. Commercial products based on activated carbon are available as tablets (DOAC-Stop^®^ and DOAC-Remove^®^) to avoid DOAC interference in LAC testing [100,101,102,103]. Additionally, filtration techniques have become available for DOAC removal from plasma samples [104,105,106]. Incomplete DOAC effect removal has been reported in some cases, both using activate carbon and filtration techniques, as well as influence on clotting times, resulting in false-negative and false-positive LAC result [101,102,104,105]. In general, DOAC adsorption products should only be used in presence of DOAC therapy, as minor changes in clotting times around the cut-off values may lead to misinterpretation of the LAC assay in non-DOAC-treated patients [68,101]. The TSVT/ET assay described above for VKA could also be useful to investigate LAC in patients treated with direct anti-FXa inhibitors, but not for direct thrombin inhibitors [65]. The two strategies, DOAC adsorption and use of TSVT/ET to overcome anti-FXa DOAC interference were compared in a recent single-center study [107]. Results showed discrepancies between the two methods and further studies are needed to investigate whether a DOAC adsorption procedure is (non-)superior to TSVT/ET in DOAC-treated patients by a head-to-head comparison.

Information on the patient’s anticoagulation status is mandatory for adequate interpretation of results. While aPTT, PT and thrombin time should be performed before starting LAC testing to have more information on the coagulation background of the patient, this is not fully conclusive because normal aPTT and/or PT do not exclude presence of DOACs or LMWH. Whenever the test results are suggestive of LAC, but there is no knowledge or doubt on the anticoagulation status, results should be reported along with warnings on the possible false positivity because of the unknown treatment status. Key messages on LAC measurement are summarized in Table 2.

### 3.3. Alternative Assays for LAC Measurement

Besides the drawback of interference on coagulation assays, LAC measurement is labor intensive and interpretation is complicated. Further search for functional assays, or other biomarkers measured with solid-phase immunoassay as alternatives for the established LAC assays should be encouraged.

Interest in thrombin generation assays (TGAs), reflecting a significant part of the coagulation system with more information compared to clotting time based assays, is growing. TGAs measure thrombin formation in plasma after addition of tissue factor and phospholipids. Contrarily to classical coagulation assays, both procoagulant and anticoagulant processes are dynamically investigated, resulting in a thrombogram with multiple derivate parameters reported [108]. TGAs can be used to assess hypo- and hypercoagulability, including APS [109,110]. TGAs show high sensitivity for detection of LAC-positive patients, and could potentially quantify LAC potency in a single assay [111,112,113]. Unfortunately, TGAs are labor intensive, poorly standardized so far, and not robust enough to include in routine setting [44,110]. Recent recommendations on how to perform TGAs may help in harmonization between methods, and support the application of TGAs in patient diagnosis and management, also in APS [110]. Recently, automated systems were introduced on the market with higher potential of use in routine practice because of reduced laboratory technician hands-on time, and decrease in inter-laboratory variation. Automated TGAs may provide more information based on phospholipid-dependent testing in one assay, compared to the multitude of assays needed for LAC detection today [108].

Antiphosphatidylserine/prothrombin antibodies (aPS/PT) measured by solid-phase assays have been investigated as surrogate marker for LAC (see further on).

## 4. Anticardiolipin and Anti-β2-Glycoprotein I IgG/IgM Antibodies

### 4.1. Choice of Assay

aCL and aβ2GPI are detected by solid-phase immunoassays, traditionally ELISA. The APS classification criteria indicate the measurement of aCL and aβ2GPI by standardized ELISA [36]. Since the publication of the Sydney laboratory criteria, alternative detection techniques for aPL testing such as chemiluminescent, fluorescence enzyme, and multiplex flow immunoassays are available [44]. In the 2010s, testing of aCL and aβ2GPI on (semi-) automated analyzers became commercially available and is increasingly used in diagnostic laboratories, but automated systems are not universally available. Compared to traditional manual ELISA methods, newer techniques apply consistent protocols, are easier to use, show less inter-laboratory variation for the same method, and are less prone to inter-operator variation [114,115,116].

In essence, assays used for aCL and aβ2GPI detection are based on the same immunoassay principle (Figure 1). An antigen (cardiolipin/β2-glycoprotein I) is coated on a solid phase (polystyrene cups, magnetic particles, microbeads or membranes) to which the antibody from plasma or serum can bind, if present. Reagents contain anti-human IgG or IgM antibodies bound to a conjugate that can bind to the Fc part of antibodies from the patient on the solid phase. Consequently, by conversion of a substrate through the conjugate, a reaction (color, chemiluminescent or fluorescent) will occur, which is measured by a detector (Figure 1). Comparison of the signal with a calibration curve quantifies the antibody titer. Assays differ in solid phase, detection principle, coating, source of antigens and antibodies, blocking agents to prevent non-specific binding, dilution protocol, calibration, and units [114,117]. A large variety of assays is commercially available. Selection of an adequate aCL assay, that is β2-glycoprotein I dependent, is important [44,117,118,119], since it increases the specificity for clinically relevant aCL antibodies compared to co-factor-independent aCL [120]. In aβ2GPI assays, β2-glycoprotein I is directly coated on the solid phase [117]. Overall, β2-glycoprotein I-dependent aCL assays and aβ2GPI assays have similar clinical sensitivity/specificity and a good correlation between both is observed [115,117,121].

Large heterogeneity in assay techniques, reagents, and calibrators leads to high inter-assay variability. Variability is observed both in qualitative (positive/negative) and quantitative (antibody titer) interpretation of aCL and aβ2GPI results, especially between ELISA and automated platforms using newer techniques such as chemiluminescence [117,122,123,124,125]. While agreement between commercially available immunoassays is poor for individual aCL and aβ2GPI IgG or IgM detection, assays showed comparable clinical accuracy when all criteria solid-phase aPL were determined [122]. Because of the variability between platforms, different aPL should be measured using the same solid phase platform to avoid unexpected discrepancies between aCL and aβ2GPI. It is recommended to perform patient follow-up testing within the same laboratory as platforms cannot be used interchangeably. On the other hand, when aPL results are negative in patients with high clinical suspicion for APS, it could be considered to retest aCL and aβ2GPI in another laboratory, using a different platform or assay.

IgG aCL and aβ2GPI are more strongly associated with thrombosis and obstetric morbidity compared to the IgM isotype, independent of the solid-phase assay used [126,127]. Based on a multicenter study, a multivariable logistic regression analysis of aPL demonstrated that IgM positivity was independently associated with obstetric morbidity and not with thrombotic APS. However, addition of IgM to LAC and IgG aPL did increase odds ratios for thrombosis. These results suggest that IgM aCL and aβ2GPI should be tested in first-line evaluation of suspected obstetric APS, but could be evaluated as second-line test for risk assessment of thrombotic APS patients [126]. Therefore, both isotypes, IgG and IgM, are included in the laboratory criteria [44].

### 4.2. Analytical Procedure

In analogy with LAC testing guidelines, the ISTH-SSC published guidelines for detection of aPL with solid-phase assays, focusing on the analytical aspects [118].

#### 4.2.1. Routine Implementation

Solid-phase assays need to be evaluated before implementation in routine practice. Because of the substantial inter-assay variability and lack of a gold standard, direct comparison of assays is not advised and local validation of its performance should be based on clinical and analytical criteria. Preferably, the association between solid-phase aPL results and clinical manifestations is evaluated [44,118]. Unfortunately, this is often not feasible because of the low incidence of APS. Analytically, precision needs to be evaluated and checked against the assay performance specifications, especially around the cut-off value. The ISTH guidelines recommend a between-run imprecision of <20%, preferably <15%, for manual ELISAs and <10% for assays performed with automated platforms [118].

Serum or platelet-poor plasma can be used for aCL and aβ2GPI testing, provided that the assay specifications and cut-off values are verified for the sample type. Use of platelet-poor plasma for LAC testing is required to avoid false-negative results due to interaction of platelet-phospholipids and aPL. By extension, this reasoning could be made for solid-phase assays as well when using plasma. Therefore, current ISTH guidelines advise on use of serum (no specification to be platelet poor) or platelet-poor plasma (specified as <10,000 thrombocytes/µL) [25].

Need for duplicate sample and quality control analysis in the same run is dependent on assay performance characteristics [116,118]. Duplicate testing is especially recommended for manual ELISAs or if inter- and intra-run imprecision of the assay is >10% [118]. In each run, internal quality control material needs to be analyzed at relevant titer levels. Preferably, these controls are reagent kit independent to control for inter-lot reagent variability [118,125].

#### 4.2.2. Calibration

Calibration curves need to be determined in every ELISA run, or for every reagent lot in automated systems to reliably determine aPL titers. Recalibration can be warranted based on internal quality control results. Each calibration should be evaluated, and rejected when not meeting the manufacturer’s requirements or when the correlation coefficient between test values and target values is beneath 0.90 [118]. There is no uniformity in reference material for assay calibration. Manufacturers provide a variety of calibrators, not always traceable to a primary standard, to use for routine calibration procedures [118,121,128]. In an attempt to standardize quantification of aCL results, polyclonal patient-derived calibrators (or standards) for aCL were developed by Harris et al. in the 1980s [129]. They determined the concentration of a dilution series of affinity-purified aCL with ELISA. The concentration was expressed in IgG phospholipid units (GPL) and IgM phospholipid units (MPL) for aCL IgG and IgM, respectively, where 1 unit stands for 1 µg/mL. Subsequently, sets of calibrators were prepared by diluting a mixture of high positive aCL patient samples with different volumes of normal serum. The concentration for each calibrator was calculated, based on the measured concentration of the undiluted mixture with high aCL level [130]. These primary calibrators are known as “Harris standards” or “Louisville standards” and subsequent generations, matched with the original calibrators, are available as lyophilized product [130,131]. Alternatively, monoclonal antibody (MoAb) standards for aCL and aβ2GPI IgG (HCAL) and IgM (EY2C9) were developed, called “Koike standards” or “Sapporo standards” [132]. Primary standards are mostly used by assay manufacturers to construct secondary calibrators, which should be mentioned in the assay specifications. MoAbs have the advantage of higher reproducibility between batches and theoretical infinite production capacity, although they do not mirror the polyclonality of antibodies encountered in APS patients [131,133]. Converting MoAbs concentrations in GPL/MPL units is possible for aCL, but is not always performed [133]. aCL results can only be reported in GPL/MPL units if validated against the original Harris standards, while no international unit is available for aβ2GPI testing and results can be expressed in a plethora of units (e.g., IU/mL, U/mL, SGU, SMU, µg/mL, G units, M units, GAU/mL, and MAU/mL) [121,133]. Ideally, all manufacturers use the same calibration material. Therefore, efforts are undertaken to develop new monoclonal and polyclonal standards for both aCL and aβ2GPI aiming to create WHO standards with IU/mL as the universal unit [133,134].

#### 4.2.3. Cut-Off Values

Current APS classification criteria consider detection of aCL IgG or IgM to be significant if present in moderate to high titer, measured with a standardized ELISA. Moderate to high titer is defined as > 40 GPL or MPL, or > 99th percentile based on a reference population [36,39]. Detection of a significant aβ2GPI level is defined by titers > 99th percentile [36]. Choice of 40 GPL/MPL as aCL cut-off was based on studies demonstrating that aCL IgG titers > 40 GPL correlated better with APS-related characteristics compared to lower positive titers [135,136]. However, there can be a striking difference between 40 GPL/MPL and the 99th percentile for aCL [117,137,138]. High inter-assay variability seems to make it impossible to advise one general numeric threshold for classifying solid-phase aPL titers as “moderate to high” and the ISTH-SSC does not advise using 40 GPL/MPL as a cut-off. It is recommended to calculate a laboratory-specific cut-off value for positivity based on a non-parametric 99th percentile of at least 120 reference individuals [118]. Outlier rejection with the Dixon/Reed method is advised to avoid overestimation of cut-off values [128]. Transference of manufacturers’ cut-offs after verification on 20 or more reference individuals is a valid alternative if the manufacturer’s cut-off is calculated on a large enough reference population and an adequate statistical methodology was applied [73,118]. Each aCL and aβ2GPI result above the cut-off has to be reported as positive, accompanied with the numeric value and the in-house cut-off value [118].

Studies showed that higher titers of IgG aCL, but not IgM, are more associated with clinical APS events compared to lower titers [139,140]. Interpretation of antibody titers as “negative” (lower than cut-off), “low” (between cut-off and 40 GPL/MPL), “medium/moderate” (between cut-off and 40 GPL/MPL), and “high” (above 80 GPL/MPL) has been suggested, but is currently not recommended due to a lack of numerical agreement between assays [44,118,130,141]. External quality control programs show that classification of aPL results into ranges of low/moderate/high differs between platforms, and users ascribe a different classification to an identical numerical test result [125]. On the other hand, semiquantitative interpretation can be useful for the clinician and could improve the interpretation of IgG aCL and aβ2GPI across laboratories [39,142,143]. We have demonstrated that the likelihood for thrombotic and obstetric APS is higher in patients with moderate/high IgG aCL and aβ2GPI titers compared to low positive titers. Likelihood for APS did not clearly increase with higher IgM aCL or aβ2GPI titers. We suggest that semiquantitative interpretation of aPL may be useful for the IgG isotype, but not for IgM, provided that assay-specific thresholds are defined to establish positivity ranges (low-moderate-high) [124]. Harmonization of semiquantitative ranges could be achieved by paired analysis of standard material or well-characterized patient populations in both the new technique and a standardized ELISA with traceability to the original Harris standards [124].

### 4.3. Interferences

Interference in immunological testing is assay dependent and known interferents should be specified in the product insert. If possible, the highest concentration not causing significant bias should be stated by the manufacturer, especially for rheumatoid factor and hemolysis/icterus/lipemia [118,144]. Presence of IgM rheumatoid factor can cause false-positive aCL IgM and aβ2GPI IgM results [145]. Contrary to LAC assays, testing of aPL with solid-phase immunoassays is not subject to analytical interference by acute-phase reactants or anticoagulation therapy. However, as for LAC, a transient increase in aCL and aβ2GPI is also observed in infectious diseases and other inflammatory conditions, stressing the importance of repeat aPL testing after >12 weeks [38,118,146]. Table 3 summarizes the key messages on aCL and aβ2GPI measurement.

## 5. Interpretation of Antiphospholipid Antibody Tests

### 5.1. Patient Selection

APS is a rare disease with an estimated yearly incidence of 2 per 100,000 persons per year and a prevalence of 50 per 100,000 with similar incidence and prevalence in both sexes [147]. The prevalence of aPL positivity in the general population is unknown, but it is thought to be at least 10-fold higher than the prevalence of APS. Indeed, aPL can circulate in healthy individuals or patients with underlying autoimmune disease without ever developing the clinical phenotype of APS [148]. In clinical practice, testing for aPL should be limited to patients where APS is suspected—typically in young patients (<50 years) with thrombosis, thrombosis at unusual sites, unexplained recurrent thrombosis, patients with severe pre-eclampsia or HELLP syndrome. Additionally, when thrombosis or obstetrical morbidity is associated with other APS manifestations such as livedo, presence of autoimmune disease, prolonged aPTT e causa ignota, or mild thrombocytopenia, testing for aPL should be included in diagnostic workup [25,39,149,150]. Identifying patients with APS is important because specific treatment and prophylactic strategies are recommended, for instance avoidance of DOAC therapy for secondary thromboprophylaxis in some patient populations [39,82,150,151]. aPL testing can also be indicated for diagnostic evaluation of SLE as part of its diagnostic criteria or for thrombotic risk assessment in SLE patients [151,152].

### 5.2. Antibody Profiles

Positivity in one of the criteria aPL (LAC, aCL IgG, aCL IgM, aβ2GPI IgG, and aβ2GPI IgM) is sufficient for diagnosing APS in presence of a clinical criterion [36,44], although antibody isotype, titer and number of positive tests also give useful information for thrombotic and obstetric risk stratification [44]. Combined interpretation of different aPL as antibody profiles was suggested to identify patients as high risk, compared to individual aPL evaluation for diagnosis [36,153]. aPL profiles are defined as “single positive” (only one isolated aPL positive), “double positive” (aCL and aβ2GPI positive, LAC negative), and “triple positive” (LAC, aCL, and aβ2GPI positive). Studies demonstrated that triple positivity was associated with a high risk of initial and recurrent thrombotic events [154,155,156,157,158]. In asymptomatic aPL carriers, both double and triple positivity was a risk factor for developing thrombotic events, but single aCL or aβ2GPI positivity was not [154]. Furthermore, in women diagnosed with APS, triple positivity was identified as the highest risk factor for adverse pregnancy outcome, together with history of thrombosis [159]. In most cases, the high-risk triple-positive profile is confirmed after 12 weeks, making it a reliable estimate for initial risk evaluation [158,160]. In a recent meta-analysis, no evidence was observed to assume that isolated IgG aβ2GPI can predict clinical APS manifestations, while it is often considered as the most pathogenic of all aPL [161]. Clinical relevance of single aCL positivity is doubtful, as this may represent presence of non-pathogenic antibodies, not directed to β2-glycoprotein I, but to cardiolipin itself [114]. However, a recent study observed a comparable high risk for thrombosis in asymptomatic carriers with isolated aCL in absence of underlying autoimmune disease and carriers with the triple positivity profile [158]. Long considered as low risk, the value of persistent single LAC in APS is growing [162]. Isolated LAC activity could be partly explained by presence of β2-glycoprotein I-independent aPL, such as aPS/PT [162,163]. Furthermore, due to the inter-assay disagreement, individuals can be negative for all solid-phase aPL in one platform, but not in another one, which might partially explain the phenomenon of single LAC positivity [122,164]. Additionally, single LAC positivity was illustrated as an independent risk factor for myocardial infarction, ischemic stroke, and adverse pregnancy outcome [159,165,166,167].

### 5.3. Scoring Systems

Information from aPL antibody profiles can be quantified by calculating a score for risk assessment. In 2012, Otomo et al. developed the antiphospholipid score (aPL-S) based on LAC and solid-phase aPL results in SLE patients with and without clinical APS manifestations. aPL-S was constructed with odds ratio-dependent values for clinical manifestations of each parameter individually. Variables in the aPL-S were different laboratory tests for LAC testing (aPTT, dRVVT, and KCT), aCL IgG/IgM, aβ2GPI IgG/IgM, and aPS/PT IgG/IgM with further discrimination between high and low/medium antibody titers for IgG antibodies, but not for IgM [168]. The aPL-S showed potential for prediction of thrombosis in patients with autoimmune disease, which was independently validated by others [168,169,170]. Later, alternative scores were developed including other cardiovascular risk factors (hyperlipidemia and arterial hypertension) called the global APS score (GAPSS). In this scoring system, all criteria antibodies and aPS/PT IgG/IgM were included, but no differentiation was made between isotype and titer [171], potentially reducing the use of available laboratory stratification power. Both GAPSS and adjusted GAPSS (without aPS/PT) showed potential for assessing obstetric and thrombotic risk [172].

## 6. Other Antiphospholipid Antibodies

### 6.1. IgA Anticardiolipin and Anti-β2-Glycoprotein Antibodies

In contrast to IgG and IgM aCL/aβ2GPI, IgA antibodies are not included in the current APS criteria as the role of IgA aCL and aβ2GPI in APS was, and still is, not clear [36,44,118,173,174,175]. Recent studies show that aCL and/or aβ2GPI IgA are associated with thrombosis and pregnancy morbidity [176,177,178]. Yet, addition of IgA antibodies to the conventional aPL panel is not recommended because of high overlap with current criteria aPL [176,178]. Clinical utility in SNAPS patients is debated as reports demonstrating significant association between isolated IgA aCL/aβ2GPI and clinical APS manifestations are limited [114,176,178,179]. Various solid-phase assays using different techniques such as ELISA and chemiluminescent immunoassay (CLIA) are commercially available for measuring IgA aPL. Regarding the analytical performance of IgA assays, a head-to-head comparison of four IgA assays showed variation in detecting IgA aCL and/or aβ2GPI with positivity ranging from 13% to 19%. Within an obstetric APS population, discrepancy was even higher, with positivity for aCL and/or aβ2GPI IgA ranging from 16% to 34% [176]. Other studies also showed standardization issues for IgA aPL assays [180,181,182], with one study reporting variable qualitative agreement (none to excellent agreement) for aβ2GPI IgA, depending on the assays compared [181]. Routine IgA aCL/aβ2GPI measurement is not recommended as added value of IgA aPL to the current laboratory criteria for diagnosis and risk stratification of APS is limited and analytical variation is high with a lack of standardization across assays.

### 6.2. Antiphosphatidylserine/Prothrombin Antibodies

Multiple studies have demonstrated a significant relationship between occurrence of thrombotic events or obstetric morbidity and aPS/PT IgG/IgM. However, aPS/PT studies are very heterogeneous and should be interpreted with care [183,184]. aPS/PT might, at least partially, explain the phenomenon of SNAPS. Recent studies observed aPS/PT positivity in up to 50% of SNAPS patients [185,186], but representativity is limited due to small sample sizes. In contrast, another study investigating consecutive patients observed aPS/PT positivity in 3% of SNAPS patients [187]. aPS/PT can also be applied as a variable in aPL scoring systems for risk assessment strategies, although formulas with and without aPS/PT have comparable clinical performance [172]. According to some studies, aPS/PT positivity and LAC is strongly correlated, with aPS/PT being positive in 77–100% of single LAC-positive individuals [163,188], although we have reported presence of aPS/PT IgG and IgM in only 7% and 11%, respectively, of patients with isolated LAC [162]. Some groups suggest that aPS/PT could be used as surrogate marker for LAC, especially for anticoagulated patients where LAC measurement is difficult [189,190,191]. While this would overcome the burden of complications accompanied with LAC testing, more studies are needed in large cohorts of APS patients to confirm this association. For now, it is unclear whether aPS/PT has additional value in APS diagnosis or risk stratification, compared to the current criteria [44,46]. aPS/PT IgG and IgM are measured with solid-phase assays but currently, only a few ELISAs are commercially available and reference standards are missing [192]. We do not recommend to routinely test for aPS/PT due to analytical restrictions, but it could provide added value for identifying false LAC negative tests in patients that show double positivity (aCL and aβ2GPI positive) [189].

### 6.3. Anti-Domain I β2-Glycoprotein I Antibodies

β2-glycoprotein I consists of five homologous domains, with domain I bearing a cryptic epitope considered to be the target of pathogenic, clinically relevant aβ2GPI [193]. Anti-domain I β2-glycoprotein I antibodies (aDI) IgG are directed against an epitope in domain I and can be measured with ELISA and CLIA [194]. Agreement between aDI and aβ2GPI is higher if both were measured with CLIA compared to studies using in-house ELISA for aDI detection [194,195,196]. Multiple studies have evaluated the role of aDI, measured with different solid-phase assays, reporting large discrepancies in clinical value [194]. It is hypothesized that the in-house ELISA measured more specifically aDI targeting the relevant cryptic epitope within domain I, while in the CLIA aDI also interacted with other domain I epitopes [194,197].

aDI IgG are often found with high antibody titer in patients with a triple-positive, high-risk aPL profile [194,195,196,197,198], and aDI positivity is associated with thrombosis and pregnancy morbidity [199,200,201]. However, several studies failed to demonstrate value for aDI as independent risk factor as they only confirm high-risk profiles and therefore added value to current laboratory criteria for risk assessment is limited [194,195,197,202]. Some authors suggested to perform aDI testing as second-line test in patients with single positivity for aβ2GPI or double positivity to identify pathogenic aβ2GPI [189,192]. Although isolated aβ2GPI might not be associated with thrombosis [161], aDI could possibly identify a pathogenic subtype. aDI is not routinely available in many laboratories as commercial assays are very limited, but if aDI testing is available, this approach seems appropriate.

### 6.4. Other Non-Criteria Antibodies

Other non-criteria antibodies are the subject of investigation for their value in APS, and especially for identifying SNAPS patients. Antibodies including those directed against phosphatidylserine (PS), phosphatidylethanolamine (PE), phosphatidic acid (PA), phosphatidylinositol (PI), vimentin/cardiolipin complex, annexin V, and annexin II are investigated. However, assays detecting these antibodies are currently poorly standardized and commercial availability is limited. Testing for antibodies against PS, PA, and PI shows potential for identifying certain obstetric SNAPS patients, but routine measurement is currently not recommended as there is limited added value compared to current criteria aPL. Antibodies against PE, vimentin/cardiolipin, and annexin V show potential to identify SNAPS patients but more research is needed to evaluate their potential role in diagnosis and risk stratification of APS [203,204,205].

## 7. Report of the Results and Information for Clinicians

All criteria aPL should be reported separately, but with an overall interpretation as the antibody profile determines the risk for clinical events in APS [44]. Antibody titer of solid-phase aPL should be reported alongside the cut-off, taking into account that numerical values are not comparable across assays [124,125]. Higher IgG titers are more associated with APS, while this is not clear for IgM [124,140], although a uniform quantitative interpretation cannot be provided across the different assays [124]. While appreciated by the clinician, reporting antibody titers in a semiquantitative manner (low/moderate/high) is currently not recommended due to the high interpretative variability across laboratories [125]. Furthermore, antibody titers around the cut-off value need to be interpreted considering the imprecision of the applied assay [118], and repeated if borderline. Reports of LAC testing should include a final conclusion on positive/negative, based on the combination of the three step procedure in the two test systems. A comment on possible interference of anticoagulants or acute-phase reactants, or use of DOAC adsorbents should be added [25]. Therefore, testing in the acute phase, pregnancy, and during anticoagulant therapy is discouraged. If performed, tests should be repeated, as false-positive and false-negative results may occur. Positive aPL results need to be confirmed anyway, after at least 12 weeks, which should be mentioned on the laboratory report [44]. Laboratory diagnosis is only one perspective in the complex picture of APS and laboratory results should always be interpreted in the clinical context. If any doubt exists on the results, a close interaction between clinician and laboratory professionals is necessary to come to an optimal diagnostic approach.

## 8. Conclusions

Laboratory investigation of aPL for diagnosis and risk stratification of APS remains a challenge, both for clinicians and laboratory staff. All currently used aPL assays have their limitations. LAC testing is a labor-intensive procedure, prone to multiple sources of interference such as acute-phase proteins and anticoagulants. Strategies for circumventing anticoagulation interference are increasingly recognized. aCL and aβ2GPI testing can be performed by an extensive range of assays and platforms, resulting in large variation of results. LAC, aCL IgG/IgM, and aβ2GPI IgG/IgM analysis should be performed and interpreted in parallel as this increases diagnostic performance. Additional biomarkers such as aPS/PT and aDI demonstrate possibilities for confirming risk estimates. Interpretative reporting of aPL results should be provided by qualified laboratory professionals.

## Figures and Tables

**Table 1:** Sydney criteria (2006) for classification of antiphospholipid syndrome.

Clinical Criteria	Laboratory Criteria
1. Vascular thrombosis - Venous, arterial or microvascular; - Confirmed by objective validated criteria; - No evidence of inflammation in vessel wall.	1. Lupus anticoagulant present in plasma on ≥2 occasions, at least 12 weeks apart.
and/or	and/or
2. Pregnancy morbidity - ≥1 unexplained fetal death ≥10th week of gestation or; - ≥1 premature birth <34th week of gestation because of: ° Eclampsia or severe pre-eclampsia ° Features of placental insufficiency; - ≥3 unexplained consecutive abortions <10th week of gestation.	2. Anticardiolipin antibody IgG and/or IgM in serum or plasma, present in medium or high titer (>40 GPL or MPL, or >99th percentile), measured by a standardized ELISA on ≥2 occasions, at least 12 weeks apart.
	and/or
	3. Anti-β2-glycoprotein I antibody IgG and/or IgM in serum or plasma, present in titer >99th percentile, measured by a standardized ELISA on ≥2 occasions, at least 12 weeks apart.

Abbreviations: ELISA, enzyme-linked immunosorbent assay; GPL, IgG phospholipid units; MPL, IgM phospholipid units.

**Table 2:** Lupus anticoagulant measurement: key messages.

**Relevance in APS diagnosis**	Functional, phospholipid-dependent coagulation assay, part of APS classification criteria. LAC theoretically detects all aPL independent of the phospholipid associated cofactor. LAC presence is a significant risk factor for thrombosis and obstetric morbidity.
**Analytical considerations**	
**Choice of assay**	dRVVT assay AND LAC-sensitive aPTT assay (or SCT): Activator: silica (preferred) or ellagic acid Low phospholipid concentration (screening)
**Analytical procedure**	Traditional assays: (1) Screening step (2) Mixing step and confirmation step if screening positive OR Paired assays: (1) Screening step and confirmation step (2) Mixing step if screening positive OR Integrated assays: Includes screen, mix, and confirm in one assay Verify that mixing step is included in the procedure
**Expression of results**	Analyze PNP in each run for every assay Calculate normalized ratio ([patient clotting time]/[PNP]) for each result or derived calculation
**Cut-off values**	In house calculation: ≥120 healthy individuals Outlier rejection with Reed method Non-parametric approach, 99th percentile No data distribution assumption Transfer of values: Cut-off values suggested by manufacturer: information on sufficient sample size and correct statistical methodology warranted; Verification with 20 or 40 healthy individuals
**Interferences**	**Consequences**
**Acute phase**	
High FVIII	False-negative aPTT, consider retest after acute phase or pregnancy.
High CRP	False-positive LAC test possible, retest after >12 weeks.
Infection/inflammation	Transient positive LAC test possible, retest after >12 weeks.
**Medication (non-anticoagulant)**	Transient positive LAC test possible, retest after cessation of medication or after >12 weeks in case of vaccine.
**Anticoagulant**	Avoid LAC testing if patient is anticoagulated. Information on anticoagulation status is mandatory.
VKA	False positive/negative LAC test possible. Interrupt VKA therapy if possible or bridge with LMWH for testing.
Heparins	No interference at therapeutic concentrations.
DOAC	Both false negatives and false positives are possible. Interrupt temporarily or use DOAC adsorption procedure.

Abbreviations: APS, antiphospholipid syndrome; LAC, lupus anticoagulant; aPL, antiphospholipid antibodies; dRVVT, diluted Russell’s viper venom time; aPTT: activated partial thromboplastin time; SCT, silica clotting time; PNP, pooled normal plasma; FVIII, coagulation factor VIII; CRP, C-reactive protein; VKA, vitamin K antagonist; LMWH, low-molecular-weight heparin; DOAC, direct oral anticoagulant.

**Table 3:** Anticardiolipin and anti-β_2_-glycoprotein I IgG and IgM measurement: key messages.

**Relevance in APS diagnosis**	Solid-phase immunoassays, part of the APS classification criteria. Important for risk stratification purposes: Double positivity and triple positivity are associated with high risk for clinical APS events; Higher IgG titers are associated with higher risk for clinical APS events than lower IgG titers. Titer of IgM is less well correlated with clinical events; IgG aCL/aβ2GPI demonstrate stronger association with thrombosis and obstetric morbidity compared to IgM; IgM aCL/aβ2GPI is independently associated with obstetric morbidity, but not with thrombotic events, although thrombotic risk is increased when positive in combination with LAC and IgG aCL/aβ2GPI; Research on value of isolated aCL or aβ2GPI IgG/IgM positivity shows conflicting results.
**Analytical considerations**	
**Matrix**	Serum or platelet-poor plasma (check assay specifications)
**Antigen in assay**	**aCL**: Cardiolipin + β2-glycoprotein I; **aβ2GPI**: β2-glycoprotein I (preferably human origin)
**Imprecision**	Manual ELISA: <20%, preferably <15%; Automated systems: <10%
**Cut-off values**	In house calculation: ≥120 healthy individuals; Outlier rejection with Reed method; Non-parametric approach, 99th percentile. Transfer of values: Cut-off values suggested by manufacturer: information on sufficient sample size and correct statistical methodology warranted. Verification with 20 or 40 healthy individuals.
**Units**	**aCL**: GPL/MPL if calibrators are matched with original Harris standards, otherwise arbitrary units; **aβ2GPI**: Arbitrary units
**Expression of results**	Numeric value based on calibration curve. Value should be reported alongside cut-off value. Results cannot be compared across laboratories or assays.
**Interferences**	**Consequence**
**Infection/inflammation**	Transient positive test possible, retest after >12 weeks.
**Rheumatoid factor IgM**	False-positive aCL/aβ2GPI IgM.
**Hemolysis/icterus/lipemia**	Assay dependent.

Abbreviations: APS, antiphospholipid syndrome; aCL, anticardiolipin; aβ2GPI, anti-β2-glycoprotein I; GPL, IgG phospholipid unit; MPL, IgM phospholipid unit.

## Data Availability

Not applicable.
